# Multicellular biomarkers of drug resistance as promising targets for glioma precision medicine and predictors of patient survival

**DOI:** 10.20517/cdr.2021.145

**Published:** 2022-06-02

**Authors:** Yuting Lu, Yongzhao Shao

**Affiliations:** Departments of Population Health and Environmental Medicine, New York University Grossman School of Medicine, New York, NY 10016, USA

**Keywords:** Drug resistance, tumor microenvironment, translational research strategy, multicellular gene correlation network, glioma, precision medicine

## Abstract

**Aim**: This study aimed to translate a known drug-resistance mechanism of long-term CSF1R inhibition into multicellular biomarkers that can serve as potential therapeutic targets as well as predictive markers for the survival of glioma patients.

**Methods**: Using existing data from a published mouse study of drug resistance in immunotherapy for glioma, we identified multicellular differentially expressed genes (DEGs) between drug-sensitive and drug-resistant mice and translated the DEGs in mouse genome to human homolog. We constructed correlation gene networks for drug resistance in mice and glioma patients and selected candidate genes via concordance analysis of human with mouse gene networks. Markers of drug resistance and an associated predictive signature for patient survival were developed using regularized Cox models with data of glioma patients from The Cancer Genome Atlas (TCGA) database. Predictive performance of the identified predictive signature was evaluated using an independent human dataset from the Chinese Glioma Genome Atlas (CGGA) database.

**Results**: Fourteen genes (*CCL22*, *ADCY2*, *PDK1*, *ZFP36*, *CP*, *CD2*, *PLAUR*, *ACAP1*, *COL5A1*, *FAM83D*, *PBK*, *FANCA*, *ANXA7*, and *TACC3*) were identified as genetic biomarkers that were all associated with pathways in glioma progression and drug resistance. Five of the 14 genes (*CCL22*, *ADCY2*, *PDK1*, *CD2*, and *COL5A1*) were used to construct a signature that is predictive of patient survival in the proneural subtype GBM patients with an AUC under the time-dependent receiver operating characteristic (ROC) of 2-year survival equal to 0.89. This signature also shows promising predictive accuracy for the survival of LGG patients but not for non-proneural type GBMs.

**Conclusion**: Our translational approach can utilize gene correlation networks from multiple types of cells in the tumor microenvironment of animals. The identified biomarkers of drug resistance have good power to predict patient survival in some major subtypes of gliomas (the proneural subtype of GBM and LGG). The expression levels of the biomarkers of drug resistance may be modified for the development of personalized immunotherapies to prolong survival for a large portion of glioma patients.

## INTRODUCTION

Glioma is an aggressive and malignant brain tumor with a poor prognosis. The traditional standard-of-care therapies (surgical removal, radiotherapy, chemotherapy, *etc*.) only slightly extend the survival of glioma patients ^[1]^. Despite the recent advances in cancer immunotherapies and targeted therapies in treating many types of cancer, only a fraction of patients developed durable responses, which indicates the common existence of intrinsic/acquired resistance to existing immunotherapies. To date, the effect of immunotherapies in treating glioma has been even more disappointing, partly due to prevalent drug resistance ^[2-4]^. To improve patient survival, it is critical to discover potential therapeutic targets and prognostic biomarkers for novel biological interventions to overcome drug resistance.

The tumor microenvironment (TME) plays a crucial role in the progression and responses to therapies ^[5]^. In addition to tumor cells (TCs), the TME also includes T cells, tumor-associated macrophages (TAMs), epithelial cells, *etc*. ^[6, 7]^ The immunosuppressive action of TAMs based on the release of anti-inflammatory cytokines within the TME could promote the proliferation of tumor cells and the subsequent drug resistance ^[5, 6, 8, 9]^. Immunotherapies are often designed to enhance antitumor capacity of the immune cells such as TAMs, and, in turn, the enhanced TAMs could attack and kill TCs. In particular, inhibition of CSF1R (by the small-molecule BLZ945 treatment) in TAMs has been a promising intervention for glioblastoma (GBM) in mice; however, persistent usage of CSF1R inhibition can lead to drug resistance in mice ^[1]^. Importantly, a well-designed mouse study published by Quail *et al*. ^[1]^ discovered and characterized the mechanism of the drug resistance to CSF1R inhibition in mice. Specifically, long-term inhibition of CSF1R in TAMs resulted in the increased secretion of IGF1 to TME and the alternative activation of TAM, which was reflected by the elevated expression level of M2-like genes. The combination of IGF1 in TME and its receptor in TCs, IGF1R, activated the downstream PI3K signaling pathways to support tumor regrowth and led to drug resistance. Based on this drug-resistant mechanism, they further identified multiple interventions, including blockage of IGF1R (by OSI906) and inhibition of PI3K pathway (by BKM120), that resulted in substantial improvement in survival in mouse studies when combined with CSF1R inhibition. However, the important findings from this mouse study of drug resistance have not been translated to human gliomas to prolong patient survival. Thus far, it is unclear whether the findings of the mice study can be successfully translated to some subtypes or all types of gliomas in humans.

Given the importance of the multiple types of cells in TME for drug resistance, it is desired to have cell-specific (immune cell and tumor cell) gene-expression data to investigate cell-specific effects and interactions between different types of cells in TME when studying drug resistance in humans. However, due to the extensive labor cost and technical challenges in obtaining cell-specific data in humans, discovering the multicellular mechanism of drug resistance directly in human trials is currently challenging. In contrast, as cell-type-specific gene expression data from mouse studies are more affordable ^[1]^, we suggest a translational study strategy that projects the multicellular results of the animal experiment to human genome to investigate drug resistance. In particular, borrowing strength from the mouse study published by Quail *et al*. ^[1]^, we can combine cell-specific mouse gene expression data with gene expression data from human bulk tissue to identify biomarkers of drug resistance and patient survival. Furthermore, as Quail *et al*. ^[1]^ also identified interventions to overcome the drug resistance in mice, any genetic biomarkers we identify would likely to be actionable targets for therapeutic intervention in human precision medicine, too.

For the purpose of developing novel treatment targets that are feasible for biomedical intervention, for convenience, we would like to select a small set of genes that can adequately account for drug resistance as well as patients' survival. However, response and resistance to an intervention typically involve a great number of genes and pathways in addition to population heterogeneity. In practice, it is hard to decide which genes are biologically more important than others, given the vast number of genes involved, and it is generally challenging to distinguish driver genes from passenger genes based on cross-sectional gene expression data. To the best of our knowledge, there is no known method that can efficiently identify biomarkers of drug resistance with high predictive accuracy for patient survival. Given that biological pathways involve the cooperation of clusters of highly correlated genes, we used gene correlation network analysis and gene-set enrichment analysis to detect biologically important gene clusters. Focusing on gene clusters in important pathways can borrow strength from existing biological knowledge based on independent studies; thus, it should be more likely to determine genes with driver effects and avoid the abundance of false positives, compared to the common approach of focusing on the analysis of individual genes with top $ P $-values. Moreover, important and well-connected genes in gene networks are generally sparse ^[10, 11]^; thus, constructing weighted gene networks reflecting such sparsity would be desirable. Moreover, regularized Cox regression models that account for the sparsity of important genes in correlation networks can be used to further shrink the number of candidate genes in order to form a compact gene set predictive of patients' survival.

In cancer research, the "one treatment for all patients" approach is generally impractical given various heterogeneities associated with cancers. For precision medicine, it is desired and more practical to find an effective and suitable treatment strategy for each particular subtype of cancer and subgroup of patients. Furthermore, it is important to identify treatment targets and biomarkers that have high prognostic accuracy for each specified subgroup of patients in order to develop novel personalized treatments including overcoming drug resistance in existing therapies. Indeed, complex diseases are often classified into subtypes characterized by the difference in histology and pathology. In particular, gliomas are usually classified into two major categories according to the World Health organization (WHO) grading: lower-grade gliomas (LGG; WHO grade II and III gliomas) and glioblastoma multiforme (GBM; WHO grade IV gliomas). GBM can be further divided into four subtypes based on their gene expression profile: classical, mesenchymal, proneural, and neural ^[12]^. Due to the heterogeneity in histology and gene expression, drug resistance for different types of patients can be due to many different biological mechanisms involving many different pathways. Given the poor overall survival of GBM and gliomas currently, it is very valuable if we can identify a particular subgroup of patients that may benefit from interventions based on the identified drug-resistant targets or pathways.

In this study, we used a translational research strategy to identify biomarkers of drug resistance as targets for precision medicines for gliomas in humans. Beginning with results and data from an existing mouse study ^[1]^, we compared the gene expression levels between drug-sensitive and drug-resistant mice to obtain differentially expressed genes (*DEGs*) in TAMs and TCs, respectively, which were then translated to human homolog. Because the mouse study was conducted on mice initiated with the proneural subtype of GBM tumor, we hope the findings of the mouse study can be translated to the proneural type GBMs in humans. Thus, our subsequent analysis will first be conducted on the proneural type GBM subjects. Next, weighted gene correlation networks of drug resistance were constructed in TAMs and TCs, using the expression data of humans and mice, respectively. Then, we performed concordance analysis to compare human networks to mouse networks within each cell type and performed enrichment analysis to get the biologically important gene clusters (indicating pathways), from which candidate genes were selected according to their importance and individual predictive capacity for patient survival. Lastly, integrating the findings of M2-like genes and PI3K pathways identified in the drug-resistance mouse study, we applied regularized Cox regression models to get a small set of genetic biomarkers. For precision medicine, it is important to identify some major subgroups or subtypes of gliomas such that expression levels of the identified molecular biomarkers of drug resistance can predict population survival rates of human glioma patients, and ideally, the expression levels of the biomarkers can be modified to prolong survival of a large portion of patients. Towards this end, time-dependent ROC curves, corresponding AUCs, and Kaplan-Meier (KM) curves were generated to demonstrate the predictive performance of the identified genetic biomarkers in the proneural subtype of GBM, non-proneural type of GBM, and LGG patients, respectively. The identified genetic biomarkers showed high AUCs at two years in the proneural subtype of GBM, indicating good predictive performance of the identified signature. Importantly, the signature developed using the proneural type mouse study had poor predictive power of survival in non-proneural subtypes of GBM, suggesting that different mechanisms and therapeutic targets should be considered for different subtypes of glioma. We also discuss the identified biomarkers as potential treatment targets to overcome drug resistance.

## METHODS

### A translational strategy to identify predictive biomarkers of drug resistance and patient survival

**Figure 1:**
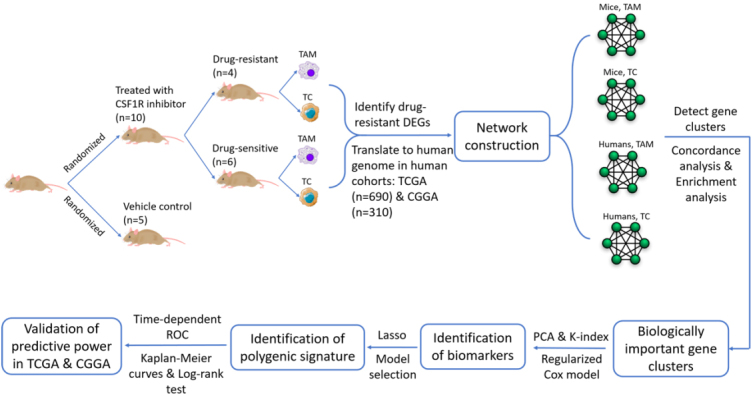
Flowchart of the translational study design. TAM: Tumor-associated macrophages. TC: Tumor cells. DEGs: Differentially expressed genes. TCGA: The Cancer Genome Atlas. CGGA: Chinese Glioma Genome Atlas. PCA: Principal component analysis. ROC: receiver operating characteristic.

Since obtaining cell-specific gene expression data in human brains is a challenge and such cell-specific mouse data are available from the study by Quail *et al*. ^[1]^, we introduced a translational study design that borrows strength from the mouse cell-specific data to identify biomarkers of drug resistance in humans. First, we identified and translated DEGs between drug-resistant (Reb) and drug-sensitive (Ep) mice to humans in TAMs and TCs, respectively. Then, weighted gene correlation networks were constructed, and gene clusters were detected for TAMs and TCs in mice and human patients, respectively. By comparing mouse networks to human networks via concordance analysis, biologically important gene clusters were selected from the highly concordant gene clusters, integrating the result from enrichment analysis. Next, to discover therapeutic targets of gliomas that may be actionable in future intervention, we reduced the number of candidate genes using principal component analysis (PCA) and K-index based on the biologically important gene clusters. In addition, since M2-like genes and PI3K pathway-related DEGs were indicated to be associated with drug resistance in mice ^[1]^, they were combined with genes selected from the biologically important gene clusters to construct predictive signatures using regularized Cox regression models. Finally, the performance of the identified predictive signature was examined by KM analysis and time-dependent ROC curves. The entire workflow is shown in Figure 1. More details are described in the following subsections.

### An existing randomized mouse study for drug resistance in gliomas

To investigate the biological mechanism of drug resistance to the CSF1R inhibition of TAMs, a randomized study of mice with gliomas was conducted and reported by Quail *et al*. ^[1]^. The CSF1R inhibition treatment is aimed at enhancing immune capacity of the tumor-associated macrophage (TAM) so that the treated TAMs can more effectively kill glioma cells or inhibit tumor growth. There were two randomized groups of mice in the study conducted by Quail *et al*. ^[1]^: the treatment naïve or vehicle group (Veh) and the treatment group or CSF1R inhibition group. The treatment group was divided into two subgroups: the group of treated mice that had durable treatment response, called the drug-sensitive or endpoint (Ep) group, and the group of treated mice that had tumor regrowth after short-term treatment response, called the drug-resistant or rebound (Reb) group. There were five Veh samples, six Ep samples, and four Reb samples with available gene expression data (RNA-seq data) for both TCs and macrophages (TAMs). The RNA-seq data of the 15 samples were selected for subsequent analyses, and the data could be downloaded from the Gene Expression Omnibus (GEO) website (https://www.ncbi.nlm.nih.gov/geo/) under the accession number GSE69104. By comparing gene expressions in TAMs of the treated and untreated (Veh) groups, we could identify differentially expressed genes (DEGs) that are modifiable by the CSF1R inhibition treatment. We could also construct gene networks in TAMs that are modified by the treatment. Furthermore, we could construct correlation gene networks for the treated mice by contrasting the drug-resistant (Reb) and drug-sensitive (Ep) groups in TAMs and in TCs, as discussed in subsequent sections.

### Human glioma cohorts

To translate the identified differentially expressed genes (DEGs) and candidate markers of the drug resistance in mice to human glioma patients, two independent human glioma cohorts, The Cancer Genome Atlas (TCGA) database (https://cancergenome.nih.gov/) and Chinese Glioma Genome Atlas (CGGA) database (http://www.cgga.org.cn/), were prepared for the subsequent gene network construction, gene cluster detection, and survival modeling. After matching the clinical information with the gene expression data (RNA-seq data) for each patient, a set of 690 samples (GBM: $ n = $ 165; LGG: $ n = $ 525) from TCGA and a set of 310 samples from CGGA (GBM: $ n = $ 138; LGG: $ n = $ 172) were collected. Since the mice were initiated with tumors from the proneural subtype of GBM, the subsequent analyses including network construction and signature identification were mainly performed in GBM proneural patients ($ n = $ 38 in TCGA; $ n = $ 30 in CGGA). In general, the TCGA dataset was used as the training dataset for the identification of the prognostic signature, and the CGGA dataset was used as an independent testing set to validate the predictive power of the polygenic signature.

### Differential gene expression analysis in mice and translation to human homolog

Differential gene expression analyses were conducted to compare the average gene expression level between drug-sensitive (Ep) and drug-resistant mice (Reb) for TCs and TAMs, respectively. The RNA-seq read counts data were normalized by the trimmed mean of $ M $-values method. For each gene, the expression level was modeled by the generalized linear model with a negative binomial link, and the quasi-likelihood (QL) $ F $-test was used to compare the gene expression level between the Ep and Reb subgroups. The logarithm of fold-change (logFC; to base 2) and nominal $ P $-value were calculated using the R/Bioconductor software package edgeR ^[13-15]^. The Benjamini-Hochberg false discovery rate (FDR) was used as an adjustment for multiple testing. Genes with $ | \log {\rm FC} | > 1.5 $ and FDR $ < $ 0.05 were considered as differentially expressed genes (DEGs) for TCs and TAMs, respectively. Due to the fact that human gene expressions are derived from the RNA-seq data of bulk tumor tissues, we only focused on DEGs that had the same signs of logFC in both TCs and TAMs in mice. This facilitated the interpretations of concordance of up-or downregulations of candidate DEGs in humans and mice. We then translated the selected DEGs identified in mice to human homolog using the NCBI database (https://www.ncbi.nlm.nih.gov/gene), which resulted in 818 DEGs in TAMs and 1730 DEGs in TCs.

### A network-based and translational research strategy to select candidate genes from important gene clusters

Our goal is to identify a set of key genes that has the potential as novel treatment targets to overcome drug resistance as well as is predictive of patient survival. The differential expression analyses typically discover a large number of DEGs, which makes it difficult in practice to design effective interventions for all of them in lab-based biological studies. Hence, we needed to refine the set of candidate DEGs to get a relatively smaller set of candidate genes that are indicative of drug resistance and predictive of survival. Depending on the co-expression network, DEGs can be clustered according to their intrinsic correlations. Clusters of genes may pertain to specific biological functions and have a greater impact on the outcome than single genes. In general, given a set of gene expression data, it is straightforward to construct correlation gene networks or weighted correlation gene networks, e.g., as done by Sun *et al*. ^[16]^ or He *et al*. ^[17]^. In the following sections, we discuss how to identify key genes from important clusters detected through weighted gene correlation networks (WGCNA) ^[18]^.

#### Detection of gene clusters (modules) by weighted correlation network analyses for TC and TAM in mice and human

First, we constructed the weighted correlation network using weighted correlation network analyses (WGCNA) for TAMs and TCs, in mice and humans, respectively, which resulted in four networks. For each of the networks, denote the gene expression matrix as $ X = [x_{ij}]_{n \times p} $, where $ n $ is the sample size, $ i = 1, \cdots, n $, $ p $ is the number of genes, and $ j = 1, \cdots, p $. Let $ \mathit{\boldsymbol{x}}^{\mathit{\boldsymbol{(j)}}} = (x_{1j}, \cdots, x_{nj})^{\rm T} $ denote the expression of the $ j^\rm{th} $ gene and $ \mathit{\boldsymbol{x}}_{i} = (x_{i1}, \cdots, x_{ip}) $ denote the gene expression of the $ i^\rm{th} $ subject. A correlation network is fully specified by its adjacency matrix $ A = [a_{ij}]_{n \times n} $, which is a symmetric $ n \times n $ matrix with entries in [0, 1] representing the connection strength of the $ i^\rm{th} $ and $ j^\rm{th} $ gene. The weighted adjacency $ a_{ij} $ is modeled by the power adjacency function, that is,

(1) $ a_{ij} = s_{ij}^{\beta}, $

where $ s_{ij} $ is the co-expression similarity that defined by the Pearson correlation, i.e.,

(2) $ s_{ij} = | {\rm{cor}} (\mathit{\boldsymbol{x}}^{\mathit{\boldsymbol{(i)}}}, \mathit{\boldsymbol{x}}^{\mathit{\boldsymbol{(j)}}}) | =  \left| \frac{\sum_{k = 1}^{\rm n}  \left(x_{ik} - \bar{x}^{(i)}\right) \left(x_{jk} - \bar{x}^{(j)}\right) }{\sqrt{\sum_{k = 1}^{\rm n} \left(x_{ik} - \bar{x}^{(i)}\right)^{2}\sum_{k = 1}^{\rm n} \left(x_{jk} - \bar{x}^{(j)}\right)^{2}}} \right|, $

where $ \bar{x}^{(i)}, \bar{x}^{(j)} $ are the mean expression level of the $ i^\rm{th} $ and $ j^\rm{th} $ genes. The power parameter $ \beta (\beta \geq 1) $ was chosen by applying the approximate scale-free topology criterion. Details can be found in the work of Zhang and Horvath (2005) ^[19]^.

The network was constructed once $ \beta $ was specified. Next, we detected clusters of genes that were tightly interconnected. Such clusters of genes in the WGCNA method are called modules. To group the highly correlated genes into modules, we needed to introduce a distance measure that quantified the dissimilarity between each pair of genes within a weighted correlation network. We adopted the topological overlap matrix (TOM)-based dissimilarity, which is commonly used in many applications. The TOM-based dissimilarity is defined as

(3) $ d_{ij} = 1 - \frac{l_{ij} + a_{ij}}{\min\{k_{i}, k_{j} \} + 1 - a_{ij}}, $

where $ a_{ij} $ is the weighted adjacency defined in the weighted correlation network, $ l_{ij} = \sum_{u} {a_{iu}a_{uj}}, k_{i} = \sum_{u} a_{iu} $. The hierarchical clustering dendrogram (tree) can be built with $ \{d_{ij} \}_{i \neq j}^{p} $. Dynamic branch cut method was applied to identify gene modules from the hierarchical clustering dendrogram ^[20]^. Parameters involved in the network construction and module detection were selected for each of the four networks individually (TAM network in humans, TC network in humans, TAM network in mice, and TC network in mice). The network construction and module detections were performed using the R/Bioconductor package WGCNA ^[18]^.

#### Identification of important gene clusters through concordance analysis between mouse and human modules

We identified four sets of modules from the weighted gene correlation network: modules for TAM in mice, modules for TC in mice, modules for TAM in humans, and modules for TC in humans. These modules can be viewed as "sub-networks" as they represent gene clusters in which genes are closely correlated. They may perform certain biological functions, since biological functions are rarely determined by a single gene, but rather by a set of tightly interconnected genes. In addition, the correlation networks and subsequently identified modules are based on DEGs that are differentially expressed between Reb mice and Ep mice. Thus, modules and their underlying biological functions identified in mice TAM and TC are likely to be associated with drug resistance. In each cell type, given that mouse modules and human modules share the same set of DEGs, and mice and humans are evolutionarily conserved, it would be of interest to know whether mouse modules and human modules perform similar biological functions or if the sub-networks and biological functions in mice are preserved in humans. If a mouse module and a human module do share a "sub-network", its underlying drug resistance-related biological functions should be more likely to be translated to humans. Therefore, in the same cell type, the concordance between each pair of mouse-human modules was assessed by calculating the number of genes that overlapped for each pair of mouse-human modules. Whether such overlap was due to chance alone was assessed by the Fisher's exact test. Contingency tables are reported for TC and TAM, respectively. Specifically, gene clusters from the top significantly associated mouse-human modules were selected by setting a threshold for $ P $-values. In addition to the translation of drug-resistant DEGs from mice to humans at "gene-level", the concordance analysis between mouse modules and human modules can be viewed as a "network-level" translation, which is more relevant to reflect biological functions, since biological functions are normally activated by a set of genes instead of a single gene. Thus, adding "network-level" translation could help avoid false positives while enhancing the likelihood of success of the translational approach.

#### Enrichment analysis of important gene clusters

The biological functions of the gene clusters identified by the overlaps were investigated by the gene set enrichment analyses (GSEA) using Metascape ^[21]^ (http://metascape.org), which is a widely used online tool for gene annotation and enrichment analysis integrating multiple well-known ontology sources, including the KEGG Pathway, GO Biological Processes, *etc*. Gene clusters that are enriched in biologically relevant pathways were selected for the subsequent analyses. Gene set enrichment analysis leverages existing biological knowledge drawn from independent, published studies and databases, which helps to find biologically important gene clusters that are more relevant to the clinical outcome and reduce the likelihood of false-positive findings. Key genes can be further selected from the biologically important gene clusters. In short, using GSEA produces results that are more likely to be biologically meaningful and reproducible because it integrates biological and statistical information from other existing databases.

#### Selection of a small set of candidate genes from biologically important gene clusters

While several gene clusters that are biologically important for disease progression were selected by the enrichment analysis, these gene clusters still contained too many genes chosen as biomarkers of drug resistance and targets for possible interventions. Thus, we sought an even smaller set of candidate genes that are not only functionally important and representative for each of the selected gene clusters, but also possess good predictive accuracy for the survival of human glioma patients. Accordingly, two criteria were adopted to select such candidate genes. The first criterion was about the importance of the gene within each selected cluster. The first principal component (PC) is a good summary metric for a given cluster, which is denoted as "eigengene" ^[18]^. Assuming the eigengene is a good representative for a given cluster, for each gene, its correlation with the eigengene can be used to quantify its importance within a cluster. Higher correlations indicate stronger biological importance. Thus, the eigengene of the $ q^\rm{th} $ selected cluster, denoted as $ E(q) $, was calculated by the PCA. Similar to the concept of module membership, we defined the cluster membership as the Pearson correlation between the $ i^\rm{th} $ gene and $ E(q) $, that is,

(4) $ CM_{i}^{(q)} = | {\rm{cor}} (\mathit{\boldsymbol{x}}^{\mathit{\boldsymbol{(i)}}}, E(q)) |. $

Important candidate genes would be highly correlated to E(q) and can be selected by choosing an appropriate cut-off for $ CM_{i}^{(q)} $.

The second criterion was about the predictive accuracy of survival. K-index is a commonly used metric that measures the overall concordance of a risk score and the survival, i.e., $ P(T_{1} > T_{2}  | R_{2} > R_{1}) $, where $ T_{j} $ is the survival time and $ R_{j} $ is the risk score ^[22]^. It does not depend on the censoring distribution, which makes it more general to assess the predictive power ^[23]^. Higher K-index implies better predictive accuracy. Then, the K-index of the $ i^\rm{th} $ gene was calculated by

(5) $ K_{i} = \frac{2}{n(n - 1)}\sum\limits_{s \neq t} \frac{I(R_{i, t} > R_{i, s})}{1 + \exp{(R_{i, t} - R_{i, s})}}, $

where $ R_{i, j} $ is the linear combination of the covariates obtained from the univariate Cox regression model for the $ i^\rm{th} $ gene and $ j^\rm{th} $ subject, and $ I(\cdot) $ is the indicator function. Genes that are predictive of survival would be selected by choosing an appropriate cut-off for $ K_{i} $. In our study, candidate genes were selected by setting $ CM_{i}^{(q)} > 0.7 $ and $ K_{i} > 0.55 $ for each of the selected biologically important clusters.

### Identification of the genetic biomarkers via regularized Cox regression model

As discussed in the previous section, we selected candidate genes from biologically important clusters according to cluster membership and K-index. However, the number of candidate genes heavily depends on the threshold chosen. A large number of genes can be selected when a low threshold is chosen. In addition, cluster membership and K-index are univariate methods, which do not take into account the correlation between genes within each cluster. Thus, the sparse-group lasso Cox regression model ^[24]^ was adopted to further shrink the number of candidate genes as biomarkers of drug resistance. It is a multivariate model that can account for sparsity and the correlation within clusters. Suppose $ p $ candidate genes belonging to $ m $ clusters were selected in previous steps, and their expressions for the $ i^\rm{th} $ sample are denoted as $ \mathit{\boldsymbol{x}}_{i} = (x_{i1}, \cdots, x_{ip}) $. Let $ \mathit{\boldsymbol{x}}_{i(l)} $ denote the gene expression of $ p_{l} $ genes in the $ l^\rm{th} $ group and $ \mathit{\boldsymbol{\beta}}_{(l)} $ denote the regression coefficient of $ \mathit{\boldsymbol{x}}_{i(l)} $. Then, the coefficient $ \mathit{\boldsymbol{\beta}} $ for $ \mathit{\boldsymbol{x}}_{i} $ is estimated by

(6) $ \hat{\mathit{\boldsymbol{\beta}}} = {\rm{arg}}\;{\rm{max}}_{\beta} \left\{l_{n} (\mathit{\boldsymbol{\beta}}) - (1 - \alpha)\lambda\sum\limits_{l = 1}^{\rm m} \sqrt{p_{l}} \| \mathit{\boldsymbol{\beta}}_{(l)} \|_{2}  - \alpha\lambda \| \mathit{\boldsymbol{\beta}} \|_{1} \right\}, $

where $ \alpha \in [0, 1] $ is the weighting parameter for the combination of lasso and group-lasso penalties, $ \lambda $ is the tuning parameter, $ l_{n}(\mathit{\boldsymbol{\beta}}) = \frac{1}{n}L_{n}(\mathit{\boldsymbol{\beta}}) $, and $ L_{n}(\mathit{\boldsymbol{\beta}}) $ is the log-partial likelihood function:

(7) $ L_{n}(\mathit{\boldsymbol{\beta}}) = \sum\limits_{i = 1}^{\rm n} \delta_{i} \left\{\mathit{\boldsymbol{x}}_{i}^{\rm T }\mathit{\boldsymbol{\beta}} - \log \left[ \sum\limits_{j \in R(t_{i})} \exp(\mathit{\boldsymbol{x}}_{j}^{\rm T }\mathit{\boldsymbol{\beta}}) \right] \right\} , $

where $ (t_{i}, \delta_{i}) $ is the observed survival time and censor indicator ($ \delta_{i} = 1 $ if the survival time is observed, $ \delta_{i} = 0 $ if the survival time is censored). The sparse group-lasso Cox regression was performed by the R package SGL ^[24]^.

According to Quail *et al*. ^[1]^, resistance to CSF1R inhibition was reflected by the elevated expression level of M2-like genes in TAMs and the activation of PI3K pathways in TCs. DEGs involved in M2-type activation and PI3K pathways are very likely to be associated with glioma survival. Therefore, we performed a sparse group-lasso (SGL) analysis to select genetic biomarkers from the combination of biologically important clusters and two additional groups of candidate genes: M2-like and PI3K pathway genes. $ \alpha $ was set as 0.98 for more sparsity within the cluster. The tuning parameter $ \lambda $ was determined by 10-fold cross-validation.

Moreover, to obtain a parsimonious model, we further reduced the number of biomarkers using the $ L_{1} $-Cox regression. The coefficients were estimated by

(8) $ \hat{\mathit{\boldsymbol{\beta}}} = {\rm{arg}}\;{\rm{max}}_{\beta} \{l_{n} (\mathit{\boldsymbol{\beta}}) - \lambda \| \mathit{\boldsymbol{\beta}}  \|_{1} \}, $

where the tuning parameter $ \lambda $ is determined by cross-validation. The candidate genes with non-zero coefficients were selected as the prognostic signature for glioma.

### Evaluation for the predictive performance of the constructed drug-resistant signature

The predictive accuracy of the signature identified in the previous section was evaluated in different subgroups using time-dependent receiver operating characteristic (ROC) analyses. In each subgroup, a Cox regression model was fitted using all genes in the final signature to obtain the regression coefficient $ \tilde{\mathit{\boldsymbol{\beta}}} $ in the training set (TCGA). Risk scores were calculated by $ \mathit{\boldsymbol{x}}_{i}^{\rm T }\tilde{\mathit{\boldsymbol{\beta}}} $ in both the training set and the testing set (CGGA). Given a cut-off $ c \in R $, the sensitivity and specificity at a specific time $ t $ is

(9) \begin{align*}Se (c, t) & = P(\mathit{\boldsymbol{x}}_{i}^{\rm T }\tilde{\mathit{\boldsymbol{\beta}}} > c  | \delta_{i}(t) = 1), \\ Sp (c, t) & = P(\mathit{\boldsymbol{x}}_{i}^{\rm T }\tilde{\mathit{\boldsymbol{\beta}}} \leq c  | \delta_{i}(t) = 0), \end{align*}

where $ \delta_{i}(t) $ is the censor indicator at time $ t $. The time-dependent ROC curve ^[25]^ could be plotted by connecting all the coordinates $ (1 - Sp(c, t), Se(c, t)) $ at time $ t $, and the time-dependent AUC at time $ t $ is

(10) $ AUC (t) = \int_{- \infty}^{+ \infty} Se(c, t) d[ 1 - Sp(c, t)]. $

In our study, one-, two-, and three-year time-dependent AUCs in training set and testing set were calculated by R package timeROC ^[26]^.

In addition, patients could be further divided into a high risk of death group and a low risk of death group by taking the median of risk scores as a cut-off. KM curves were generated for the high-risk and low-risk groups of patients, and the log-rank test was employed to examine the significance of the difference in the overall survival between the high/low-risk groups.

## RESULTS

### Module detection from correlation networks constructed in mouse and human

**Figure 2:**
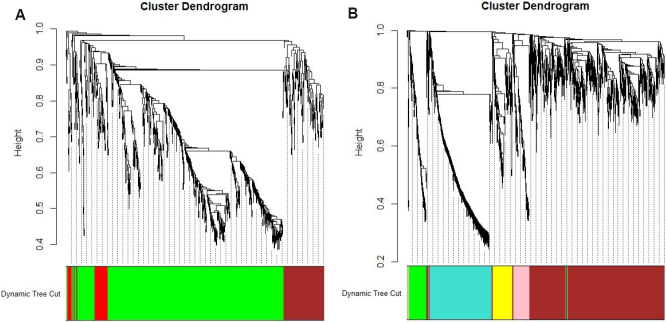
Hierarchical clustering dendrograms and modules identified in mice. (A) Dendrogram for TAM in mice. Four modules were identified (including the grey cluster, which represents genes that were not assigned to any cluster). (B) Dendrogram for TC in mice. Six modules were identified (including the grey module). Each branch refers to a gene and is marked by different colors, which represent different modules. The "height" axis refers to the value of the TOM-based dissimilarity.

Based on the translation of DEGs of drug resistance from mouse to human homolog, 818 DEGs were used to construct a correlation network for macrophages (TAMs), and 1730 DEGs were used for tumor cells (TCs), in both mice and humans. On the one hand, for the mouse TAM network, the soft threshold power parameter $ \beta $ was chosen to be 9 by applying the approximate scale-free topology criterion. Using the dynamic branch cutting method, setting the "deepSplit" parameter to be 3, "minClusterSize" parameter to be 30, and merging the highly correlated modules together, four modules were identified, as demonstrated in Figure 2A. Each branch referred to a gene and was marked by different colors, which represented different modules. Genes not assigned to any module were marked in grey. For the mouse TC network, the soft threshold power parameter $ \beta $ was chosen to be 16. Using the dynamic branch cutting method, setting the "deepSplit" parameter to be 3, "minClusterSize" parameter to be 50, and merging the highly correlated modules together, six modules were identified, including the grey module, demonstrated in Figure 2B.

**Figure 3:**
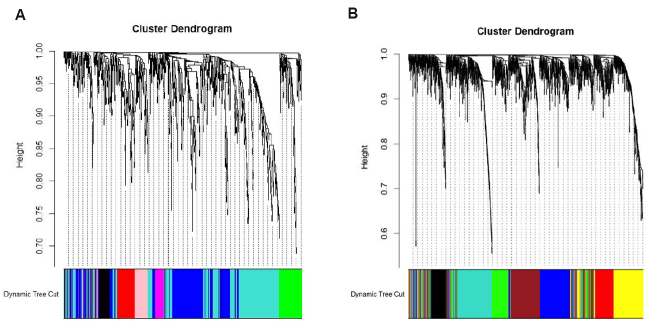
Hierarchical clustering dendrograms and modules identified in humans. (A) Dendrogram for TAM in humans. Seven modules were identified (including the grey cluster, which represents genes that were not assigned to any cluster). (B) Dendrogram for TC in humans. Eight modules were identified (including the grey module). Each branch refers to a gene and is marked by different colors, which represent different modules. The "height" axis shows the value of the TOM-based dissimilarity.

On the other hand, the human TAM network was constructed using the WGCNA method on patients classified as the proneural subtype GBM using 818 DEGs translated from the candidate genes in TAMs of mice differentially expressed between drug-resistance and drug-sensitive subgroups of mice. The soft threshold power parameter $ \beta $ was chosen to be 6 by applying the approximate scale-free topology criterion. Using the dynamic branch cutting method (by means of dissimilarity matrix of mice), setting the "deepSplit" parameter to be 3, "minClusterSize" parameter to be 30, and merging the highly correlated modules together, seven modules were identified (including the grey module), as demonstrated in Figure 3A. The human TC network was constructed on patients classified as GBM proneural using 1730 DEGs. The soft threshold power parameter $ \beta $ was chosen to be 6. Using the dynamic branch cutting method, setting the "deepSplit" parameter to be 3, and "minClusterSize" parameter to be 70, eight modules were identified (including the grey module), as demonstrated in Figure 3B.

### Selection of biologically important gene clusters from top-related human-mouse modules

**Figure 4:**
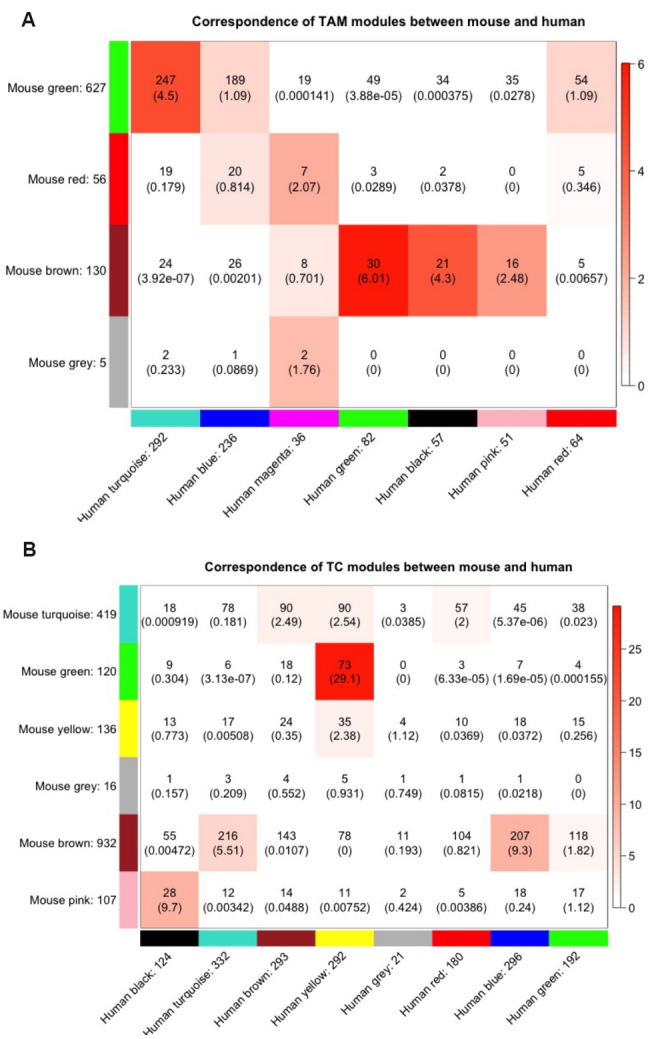
Correspondence between mouse and human modules: (A) correspondence of TAM modules between mouse and human; and (B) correspondence of TC modules between mouse and human. The human modules with their sizes were spread as columns, and the mouse modules with their sizes included were spread as rows. In each cell, the number of overlapped genes for a given pair of mouse-human modules was calculated, and the statistical significance of the overlap was tested by the Fisher's exact test. The $ P $-value is shown in the parathesis (in $ -\log_{10} $ scale). The color scale also represents the $ P $-value of the Fisher's exact test: the darker the color, the lower the $ P $-value and the stronger significance of the overlap. TAM: Tumor-associated macrophages. TC: Tumor cells.

Four modules were identified in the mouse TAM network, six modules in the mouse TC network, seven modules in the human TAM network, and eight modules in the human TC network. Since the mouse and human networks were constructed based on the same set of genes, we examined the similarities between them within the same type of cell. Thus, the contingency tables were generated to show the overlaps of each pair of mouse-human modules for TAM and TC, respectively, as shown in Figure 4A and B. The human modules with their sizes were spread as columns, and the mouse modules with their sizes included were spread as rows. In each cell, the number of overlapped genes for a given pair of mouse-human modules was calculated. Fisher's exact test was applied to test whether the overlap was statistically significant versus due to chance alone, and the $ P $-value is shown in the parentheses (in $ -\log_{10} $ scale). The color scale represents the $ P $-value of the Fisher's exact test: the darker the color, the lower the p-value and the stronger significance of the overlap.

By setting a threshold for p-value $ < $ 10$ ^{-4} $, in Figure 4A, the top three most significant gene clusters overlapped between mice and humans for TAM are mouse brown-human green (MH-TAM1), mouse green-human turquoise (MH-TAM2), and mouse brown–human black (MH-TAM3). In Figure 4B, the top four most significant gene clusters overlapped between mice and humans for TC are mouse green-human yellow (MH-TC1), mouse pink-human black (MH-TC2), mouse brown-human blue (MH-TC3), and mouse brown-human turquoise (MH-TC4). Genes in each of the seven clusters are highly correlated in both mice and humans, which makes the drug resistance more likely to be translated to humans from mice.

**Figure 5:**
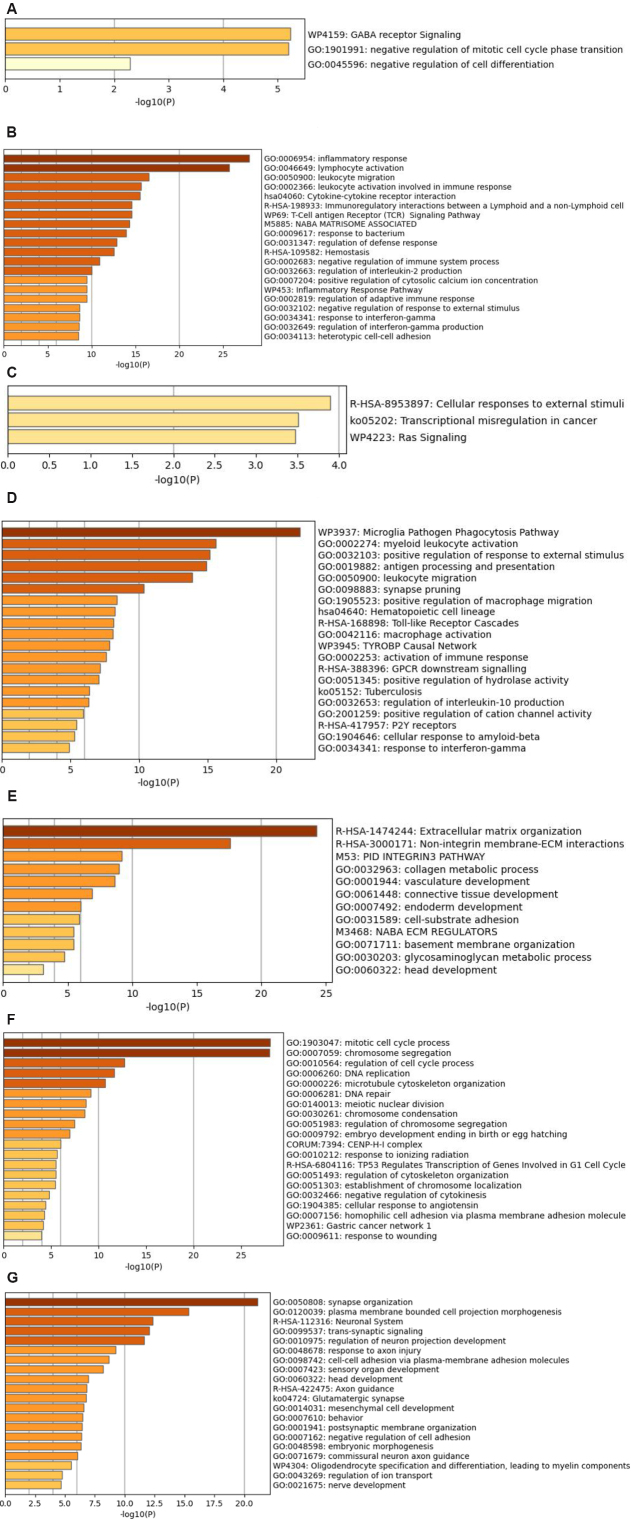
Functional enrichment analyses for the seven gene clusters overlapped between mouse and human: (A) mouse brown-human green in TAM (MH-TAM1); (B) mouse green-human turquoise in TAM (MH-TAM2); (C) mouse brown–human black in TAM (MH-TAM3); (D) mouse green–human yellow in TC (MH-TC1); (E) mouse pink-human black in TC (MH-TC2); (F) mouse brown–human blue in TC (MH-TC3); and (G) mouse brown-human turquoise in TC (MH-TC4).

To see what biological impacts these overlaps might have, functional gene set enrichment analyses (GSEA) were performed on each of the seven clusters with significant overlaps. The results are shown in Figure 5A-G. Figure 5A-C shows that, in TAM, MH-TAM1 was enriched in GABA receptor signaling and cell cycle; MH-TAM2 was enriched in inflammatory response, lymphocyte activation, *etc*.; and MH-TAM3 was enriched in cellular responses to external stimuli. Figure 5D-G suggests that, in TC, MH-TC1 was enriched in microglia pathogen phagocytosis pathways, *etc.*; MH-TC2 was enriched in extracellular matrix organization *etc.*; MH-TC3 was enriched in mitotic cell cycle process, chromosome segregation, *etc*.; and MH-TC4 was enriched in synapse organization, neural system, *etc*. The inflammatory response, microglia pathogen phagocytosis pathways, extracellular matrix organization (ECM), mitotic cell cycle process, *etc.*, are the most significantly enriched pathways and are believed to play an important role in cancer metabolism and progression. Specifically, inflammation increases susceptibility to cancer development and facilitates all stages of tumorigenesis ^[27]^. Microglia is crucial in phagocytosing tumor cells ^[28]^. In tumor tissues, the growth and malignancy of tumors as well as the response to therapy are affected by the ECM ^[29]^. Thus, we mainly focused on the MH-TAM2, MH-TC1, MH-TC2, and MH-TC3 clusters for the subsequent identification of candidate genes and drug-resistant signatures.

### Identification of the drug-resistant biomarkers and predictive signature of survival in the proneural subtype of GBM

We wanted to effectively link expression levels of candidate biomarkers of drug resistance to the population survival rate of human glioma patients. To obtain a relatively smaller but most important candidate set of genes for the identification of drug-resistant biomarkers, cluster membership (CM) and K-index were first calculated for each gene within each of the four biologically important gene clusters among proneural GBM patients. By setting the thresholds $ CM > 0.7 $ and $ K > 0.55 $, 105 genes were selected as functionally important and predictive of survival from the four selected biologically important clusters. In addition to the 105 genes, since M2-like genes and IGF/PI3K pathways were considered important for drug resistance in immunotherapy in animal models ^[1]^, 15 M2-like and 5 PI3K pathway genes that were also differentially expressed between Ep and Reb mice were added as two additional groups of genes. As a result, 125 candidate genes were prepared for the construction of prognostic signatures.

Next, considering both the correlation and the sparsity within each cluster, a sparse group-lasso was performed on the 125 candidate genes from six groups: MH-TAM2, MH-TC1, MH-TC2, MH-TC3, M2-like genes, and PI3K-related pathways. If modules (gene clusters) were detected via a dissimilarity matrix from human data, a similar set of candidate genes would be selected. Given the tuning parameters $ \alpha = 0.98 $ and $ \lambda_{SGL} = 0.0254 $, 14 genes were selected as drug-resistant biomarkers: *CCL22*, *ADCY2*, *PDK1*, *CP*, *ZFP36*, *CD2*, *PLAUR*, *ACAP1*, *COL5A1*, *FAM83D*, *PBK*, *FANCA*, *ANXA7*, and *TACC3*. Twelve of them were also selected when modules were detected using the dissimilarity matrix from mouse data. The gene names, cell types, and related pathways/gene clusters of the selected 14 genes are summarized in Table 1. Their biological functions related to gliomas are further illustrated in the discussion.

**Table 1:** Summary for the 14 identified genetic biomarkers

**Gene symbol**	**Gene name**	**Cell type**	**Gene clusters**	**Pathways**
*CCL22*	C-C motif chemokine ligand 22	TAM	M2-like gene	Inflammatory response; response to cytokine
*ADCY2*	Adenylate cyclase 2	TC	PI3K pathway-related genes	PI3K pathways
*PDK1*	Pyruvate dehydrogenase kinase 1	TC	PI3K pathway-related genes	PI3K pathways
*CP*	Ceruloplasmin	TAM	MH-TAM2	Positive regulation of cytosolic calcium ion concentration
*ZFP36*	ZFP36 ring finger protein	TAM	MH-TAM2	Response to cytokine; leukocyte activation
*CD2*	CD2 molecule	TAM	MH-TAM2	Lymphocyte activation; positive regulation of cytokine production
*PLAUR*	Plasminogen activator, urokinase receptor	TAM	MH-TAM2	Regulation of leukocyte activation
*ACAP1*	ArfGAP with coiled-coil, ankyrin repeat and PH domains 1	TAM	MH-TAM2	–
*COL5A1*	Collagen type V alpha 1 chain	TC	MH-TC2	ECM organization; PI3K pathways
*FAM83D*	Family with sequence similarity 83 member D	TC	MH-TC3	mitotic cell cycle, *etc*.
*PBK*	PDZ binding kinase	TC	MH-TC3	mitotic cell cycle, *etc*.
*FANCA*	FA complementation group A	TC	MH-TC3	mitotic cell cycle, *etc*.
*ANXA7*	Annexin A7	TC	MH-TC3	–
*TACC3*	Transforming acidic coiled-coil containing protein 3	TC	MH-TC3	mitotic cell cycle, *etc*.
Cell type (TAM or TC) refers to the cell from which the gene was selected. Gene clusters list the gene clusters to which the gene belonged. Pathways indicate the pathways that the gene was associated with (if any). TC: Tumor cells. TAM: Tumor-associated macrophages				

For the purpose of building a parsimonious model including a small set of genes that are most likely to serve as the potential targets for developing novel treatments, the biomarkers were further reduced by fitting $ L_{1} $-penalized Cox regression model. Given the $ \lambda_{\rm{Lasso}} = 0.1450 $, five genes were finally selected for the polygenic signature: CCL22, ADCY2, PDK1, CD2, and COL5A1. Fitting a Cox regression model on the five genes (gene expression was standardized by median and IQR) with adjustment of age, the risk score (RS) can be calculated as the linear combination of the covariates by the following formula:

(11) $ RS & = 0.04203 \times \rm{Age} - 0.65889 \times \rm{CCL22} - 0.65991 \times \rm{ADCY2} + 0.40717 \times \rm{PDK1}  \\ &\quad\;  + 1.14938 \times \rm{CD2} + 0.25144 \times \rm{COL5A1}. $

Here, CCL22 is an M2-like gene. ADCY2, PDK1, and COL5A1 are all involved in PI3K-related pathways. COL5A1 was chosen from the *MH-TC2* gene clusters and is involved in an extracellular matrix organization. CD2 was chosen from the *MH-TAM2* gene cluster associated with inflammatory response. All five of the signature genes have been demonstrated in the literature to be associated with the progression and metabolism of multiple malignant tumors, including GBM. Details are further illustrated below.

### Evaluation of the predictive accuracy of the drug-resistant signature in different subgroups

**Figure 6:**
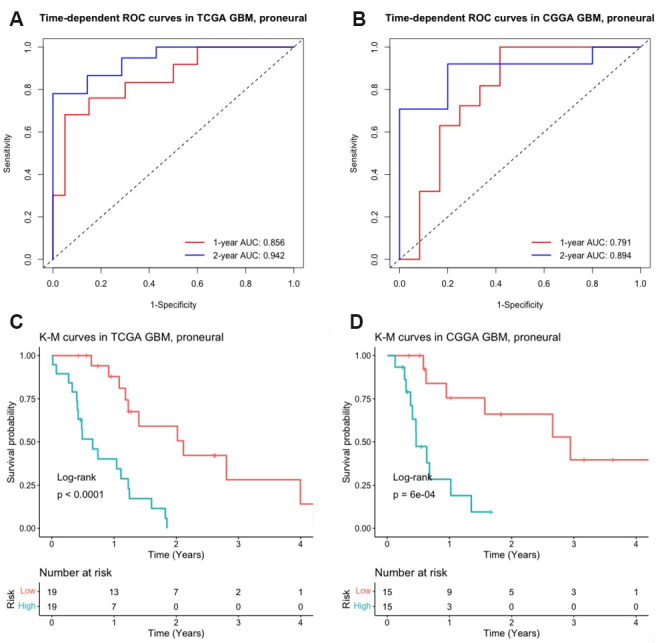
Evaluation of the predictive performance of the identified signature in the proneural subtype of GBM: (A) time-dependent ROC curves with corresponding AUCs at one and two years in training set (TCGA, $ n = $ 38); (B) time-dependent ROC curves with corresponding AUCs at one and two years in testing set (CGGA, $ n = $ 30); (C) KM curves for high-risk and low-risk patients in training set; and (D) KM curves for high-risk and low-risk patients in testing set. $ P $-values were calculated from the log-rank test. TCGA: The Cancer Genome Atlas. CGGA: Chinese Glioma Genome Atlas. ROC: receiver operating characteristic. GBM: glioblastoma multiforme. KM: Kaplan-Meier.

We first assessed the predictive performance of the identified signature in the proneural type GBM patients. Risk scores were calculated in both training set (TCGA) and testing set (CGGA) by Equation (12) with gene expressions standardized by median and IQR. Figure 6A shows the time-dependent ROC curves in the training set (TCGA). The corresponding 1- and 2-year AUCs were 0.856 and 0.942, respectively. Figure 6B shows the time-dependent ROC curves in testing set (CGGA). The corresponding one- and two-year AUCs in the independent testing set were 0.791 and 0.894, respectively, which demonstrated that the identified signature possessed a high predictive accuracy of survival in the proneural subtype of GBM patients. Moreover, K-M curves were generated for high-risk and low-risk patients classified by the median of the risk scores, as shown in Figure 6C and D. The overall survivals were significantly different between the high-risk group and low-risk group in both training and testing sets (Log-rank test: $ P < $ 0.0001 in training set and $ P = 6 \times 10 ^{-4} $ in testing sets).

**Figure 7:**
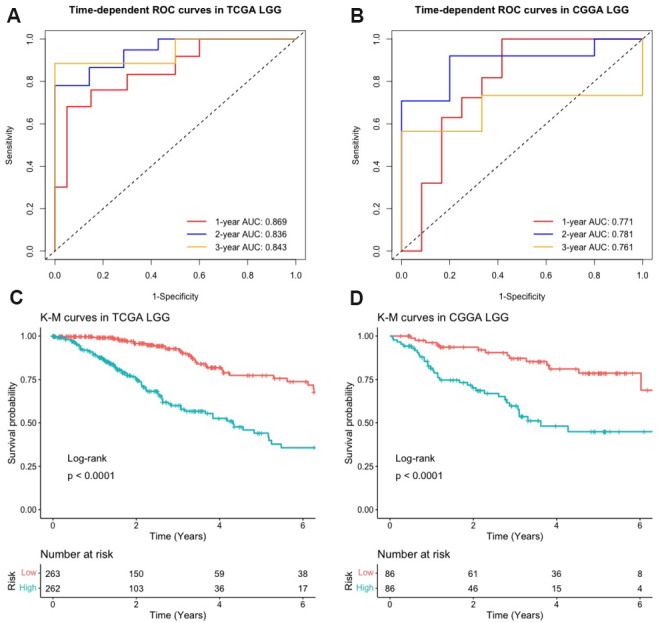
Evaluation of the predictive performance of the identified signature in LGG: (A) time-dependent ROC curves with corresponding AUCs at one, two, and three years in training set (TCGA, $ n = $ 525); (B) time-dependent ROC curves with corresponding AUCs at one, two, and three years in testing set (CGGA, $ n = $ 172); (C) KM curves for high-risk and low-risk patients in training set; and (D) KM curves for high-risk and low-risk patients in testing set. $ P $-values were calculated from the log-rank test. TCGA: The Cancer Genome Atlas. CGGA: Chinese Glioma Genome Atlas. ROC: receiver operating characteristic. LGG: lower-grade gliomas. KM: Kaplan-Meier.

Furthermore, since many LGG will eventually progress to high-grade gliomas, with the majority of them being the proneural subtype of GBM ^[30, 31]^, it would be interesting to investigate the prognostic and predictive properties of the drug-resistant signature within LGG. We therefore refitted the Cox model using the five signature genes and age in a training set of 525 LGG patients from TCGA. We validated it using an independent testing set of 172 LGG patients from CGGA. Risk scores were calculated based on the standardized expression levels. Figure 7A and B shows that the time-dependent AUC of this signature at one, two, and three years were 0.896, 0.836, 0.843 in training set and 0.771, 0.781, 0.761 in testing set. Figure 7C and D indicates that the overall survivals were significantly different between the high-risk group and low-risk group classified by the median of risk scores, as $ P $-value $ < $ 0.0001 in both training and testing sets. These results suggest that the drug-resistant signature identified in GBM proneural subtype also has good prognostic power in LGG.

**Figure 8:**
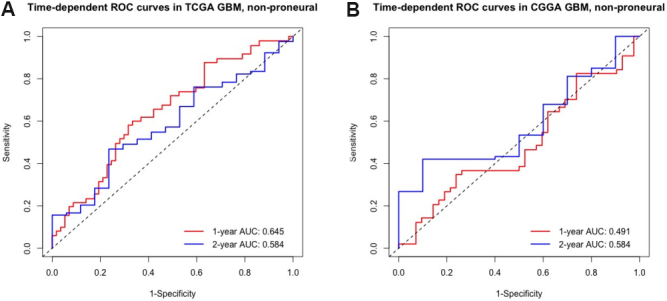
Evaluation of the predictive performance of the identified signature in non-proneural type of GBM: (A) time-dependent ROC curves with corresponding AUCs at one and two years in training set (TCGA, $ n = $ 127); and (B) time-dependent ROC curves with corresponding AUCs at one and two years in testing set (CGGA, $ n = $ 108). GBM: glioblastoma multiforme. TCGA: The Cancer Genome Atlas. ROC: receiver operating characteristic.

The mouse study reported by Quail *et al*. ^[1]^ was conducted on the proneural subtype of mice; thus, one may doubt whether the findings and biomarkers would be generalizable to non-proneural type of GBM patients. Indeed, in contrast to GBM proneural subtype and LGG, the identified candidate genes had very poor prognostic power in non-proneural types of GBM patients. To be specific, 127 non-proneural GBM patients collected from TCGA were used as the training set and 108 non-proneural GBM patients from CGGA were used as the testing set. We retrained the Cox model using the five candidate genes and age in the training set and calculated risk scores in both sets based on standardized expression levels. In the training set, the time-dependent AUCs at one and two years were only 0.645 and 0.584, respectively; in the testing set, they were 0.491 and 0.584, respectively [Figure 8]. Thus, the findings from the mouse study are not generalizable to non-proneural type GBMs, which is not surprising due to different genomic profiles for the different subtypes of GBMs.

## DISCUSSION

Glioma is the most malignant and invasive tumor that has a poor prognosis, with a median survival of GBM of only 12 months ^[12, 31]^. Despite the advances in cancer immunotherapy, patients still have limited sensitivity to current therapies, which implies a high prevalence of drug resistance. In fact, CSF1R inhibition as a treatment of glioma is being evaluated in some early-phase human clinical trials. However, some trials have been stopped or failed due to no evidence of survival improvement. The reported early-stage trials did not focus on subtypes of GBM, thus did not have adequate power to detect treatment effect in a subtype of GBMs given the small sample size. Even the likely responsive subgroups of GBM patients may have drug resistance ^[32, 33]^. Quail *et al*. ^[1]^ identified the drug resistance mechanism that the long-term inhibition of CSF1R in macrophage cells could activate IGF1/PI3K pathway in tumor cells and lead to drug resistance in mice. Therefore, it is of particular interest in identifying evidence to indicate whether the drug resistance mechanism in mice might also exist in human glioma patients, and whether some subgroups of GBM patients might have better survival using such therapies, in order to potentially improve the response and feasibility of this therapy in humans. In this study, we carried out a network-based, translational research strategy to identify potential targets for therapies with gene signatures that are predictive of survival and indicative of drug resistance to CSF1R inhibition treatment. Specifically, borrowing strength from the mouse study, we identified candidate genes that were differentially expressed between the drug-sensitive and drug-resistant mice, and translated those genes to human homologs. Then, those DEGs were used to construct weighted gene correlation networks in TAMs and TCs, for mice and the proneural subtype of GBM patients, respectively. Clusters of genes (modules) were detected from each of the networks, and biologically important gene clusters were identified as DEGs with top significant overlaps between human and mouse modules, incorporating results of gene-set enrichment analyses. The construction of weighted gene networks and detection of gene clusters in humans borrow information from the dissimilarity matrix from the mouse data to improve stability, given the lack of cell-specific gene expression data in humans. To obtain a smaller candidate gene set, functionally important and predictive genes were selected via cluster membership and the K-index from those gene clusters as well as M2-like and PI3K-related pathway DEGs. The regularized Cox regression models were then applied to further shrink the candidate gene set to obtain genetic biomarkers that are more likely to be actionable, which resulted in 14 genes (*CCL22*, *ADCY2*, *PDK1*, *ZFP36*, *CP*, *CD2*, *PLAUR*, *ACAP1*, *COL5A1*, *FAM83D*, *PBK*, *FANCA*, *ANXA7*, and *TACC3*).

Knowing the selection process, it is not a surprise that all the candidate genes selected are known to play important functional roles in cancer progression, as reported in the literature. In particular, *CCL22* is an M2-like gene, while *ADCY2*, *PDK1*, and *COL5A1* belong to PI3K pathway. According to Quail *et al*. ^[1]^, resistance to CSF1R inhibition was reflected by elevated expression of M2-like genes in TAM and activation of PI3K pathway in TC. *ZFP36*, *CP*, *CD2*, *PLAUR*, and *ACAP1* were selected from the gene cluster that was enriched in inflammatory, immune response, and regulation of cytokine pathways (MH-TAM2). Inflammation and immune responses are associated with increased susceptibility to cancer development and facilitate all stages of tumorigenesis ^[27, 34]^. Cytokines are potent but complex immune mediators and have drawn great attention to the development of cancer immunotherapies ^[35]^. *COL5A1* also came from the gene clusters that were enriched in ECM organization (MH-TC2). In tumor tissues, the growth and malignancy of the tumor as well as the response to therapy are affected by the ECM ^[29]^. *FAM83D*, *PBK*, *FANCA*, *ANXA7*, and *TACC3* were selected from the gene cluster that was enriched in mitotic cell process pathway (MH-TC3). Aberrant activities of various cell cycle proteins can lead to uncontrolled proliferation in cancer. Targeting mitotic cell cycle has been studied as a novel cancer treatment strategy ^[36-38]^. Since these gene clusters were identified from DEGs that were differentially expressed between the drug-resistant mice and the drug-sensitive mice, these pathways are likely to be associated with drug resistance.

In the literature, the 14 identified genes have been suggested as essential for the development and progression of many cancers including gliomas. Particularly, CCL22 (C-C motif chemokine ligand 22) is found in many types of human cancers and has lower expression levels in gliomas cases than in controls ^[39, 40]^. As a T cell trafficking chemokine, CCL22 attracts regulatory T cells (Treg), which could promote tumorigenesis. Inhibiting Treg trafficking in GBM may be a novel strategy to develop therapeutic interventions, which has been shown to be effective in other cancer models ^[41]^. ADCY2 (adenylate cyclase 2), which is involved in the calcium signaling pathway, may play a crucial role in the development and progression of gliomas ^[42]^. Aberrant methylation of ADCY2 is observed in many other cancers ^[43]^. PDK1 (pyruvate dehydrogenase kinase 1) is a hypoxia-inducing factor (HIF)-1 regulated gene which may promote EGFR activation that can subsequently sustain malignant progression ^[44, 45]^. By inactivating PDK1, glioma cell colony and sphere formation could be greatly inhibited, and glioma spheres would become more sensitized to temozolomide (TMZ) toxicity ^[46-49]^. CD2 (CD2 molecule) is a transmembrane molecule expressed on T, natural killer (NK), and dendritic cells and is essential for immunology ^[50, 51]^. It was found to be involved in tumor invasion and is highly expressed in breast cancer ^[50, 52]^. COL5A1 (collagen type V alpha 1 chain) was found to be related to the occurrence and progression of multiple types of malignant tumors, including breast cancer and gliomas. Recent studies found COL5A1 was positively correlated with the increasing malignancy of glioblastoma through the PPRC1-ESM1 axis activation and extracellular matrix remodeling, and it may be a potential therapeutic target for glioma ^[53-56]^. FAM83D (family with sequence similarity 83 member D) is a member of FAM83 family (including FAM83A, FAM83B, and FAM83D), which has been shown to have oncogenic potential recently. FAM83D was found to be consistently upregulated across human tumor types, including gliomas ^[57, 58]^. PBK (PDZ binding kinase) expression, which is associated with cell growth and apoptosis, DNA damage repair, immune responses, *etc*., plays an essential role in tumorigenesis and metastasis. It was found to be upregulated in GBM patients ^[59, 60]^. An *in vivo* study reported that inhibition of PBK could almost completely abolish tumor growth, which made PBK serve as a potentially promising therapeutic target for GBM treatment ^[61, 62]^. FANCA (FA complementation group A) is associated with tissue proliferation and was found to be overexpressed in many types of cancers ^[16, 63, 64]^. FANCA is essential for the function of Fanconi anemia (FA) pathway. Targeting the FA pathway may provide a novel strategy for the sensitization of solid tumors and investigation of chemoresistance in different tumor types ^[65]^. ANXA7 (annexin A7) is a ubiquitinated tumor suppressor gene ^[66]^. Loss of ANXA7 function stabilizes the EGFR protein, augments EGFR transforming signaling in glioblastoma cells, and promotes tumorigenesis ^[67, 68]^. TACC3 (transforming acidic coiled-coil containing protein 3) is often mentioned with FGFR3-TACC3 fusion, which is an oncogenic driver. FGFR3-TACC3 fusions generate powerful oncogenes that combine growth-promoting effects with aneuploidy through the activation of as yet unclear intracellular signaling mechanisms ^[69, 70]^. FGFR inhibition has shown encouraging outcomes in mouse studies ^[70]^. Targeting FGFR3-TACC3 fusion is evaluated by many ongoing early phase human clinical trials ^[70-72]^. ZFP36 (ZFP36 ring finger protein) is a well-known mRNA binding protein. In the tumor microenvironment, ZFP36 might reduce the growth and invasion of glioma cells by targeting IL-13 mRNA to inhibit the role of PI3K/Akt/mTOR pathways ^[73-75]^. CP (ceruloplasmin) serves as a prognostic biomarker in many cancers, including bile duct cancer, bladder cancer, breast cancer, *etc*. ^[76-78]^. The expression of ACAP1 (ArfGAP with coiled-coil, ankyrin repeat and PH domains 1) is correlated with immune infiltration levels in many types of cancers ^[79-81]^. PLAUR encodes the urokinase receptor (uPAR). The overexpression of PLAUR has been shown to be associated with poor prognosis in many types of gliomas, particularly in mesenchymal subtype GBM and LGG ^[82-84]^. Indeed, the 14 identified genes are more likely to reflect drug resistance and serve as potential targets since they are differentially expressed between drug-resistant and drug-sensitive mice. They might be modified in patients just as they can be modified in mice, as studied by Quail *et al*. ^[1]^.

In addition, among the 14 genetic biomarkers, five genes (*CCL22*, *ADCY2*, *PDK1*, *CD2*, and *COL5A1*) were chosen to form a prognostic signature using the $ L_{1} $-Cox regression model. The established signature has good prognostic power in the proneural subtype of GBM and LGG patients. We set TCGA as the training set for modeling and used CGGA, an independent cohort, as the testing set to validate the performance. In proneural subtype of GBM patients, the two-year AUC of this signature attained 0.89 in the testing set, which reveals the potential to build treatment targets for improved patient survival. Furthermore, as Quail *et al*. ^[1]^ also identified interventions to overcome the drug resistance in mice, any genetic biomarkers we identified here would likely to be modifiable targets for therapeutic intervention in humans. Thus, new clinical trials targeting proneural type GBMs might be developed. This drug-resistant signature also shows moderate time-dependent AUCs in LGG patients. Since many LGG will eventually progress to high-grade gliomas, with the majority of them being the proneural subtype of GBM ^[30, 31]^, it would be interesting to investigate which LGG might progress to the proneural type of GBM, and it would be of clinical importance to find out whether a novel therapy based on our candidate target genes might prevent LGG from progression to advanced stage and prolong patient survival. Consequently, using a translational network-based multicellular analysis, we linked the drug-resistance mechanism identified in mice to population-level survival rates of both the proneural type GBM and a large number of LGG patients. Importantly, the biomarkers identified from the mouse study of proneural type have a poor predictive power of survival in non-proneural GBM patients, which implies that new biological mechanisms need to be identified for the non-proneural type of GBM patients. The 14 identified biomarkers and the signature are promising targets for therapies in glioma precision medicine, or individualized treatment, because they are potentially feasible only in some subgroups of glioma patients instead of all glioma patients.

One limitation of our human study is that we only have gene expression data from bulk tissue, not knowing expression levels in TCs and in TAMs, respectively. As the cell-specific RNA-seq gene expression data will become increasingly available in the future, one might be able to construct gene networks and models using human cell-specific RNA-seq data, which would further improve the efficiency and precision of the methods we developed here to identify candidate biomarkers. In addition, with cell-specific human gene expression data on TAMs and on TCs, we would be able to model and investigate the interactions between TAMs and TCs, which is clearly important in directly investigating the mechanisms of drug resistance in humans and the identification of novel treatment targets to overcome drug resistance and prolong survival of patients.

## DECLARATIONS

### Acknowledgments

The authors would like to thank Dr. Xiaoqiang Sun and Dr. Xinwei He of Sun Yat-sen University for helpful discussions.

### Authors' contributions

Conceptualization, investigation, writing: Lu Y

Conceptualization, supervision, writing: Shao Y

### Availability of data and materials

The mouse RNA-seq gene expression data is available on Gene Expression Omnibus (GEO) website (https://www.ncbi.nlm.nih.gov/geo/) under the accession number GSE69104. TCGA glioma data can be downloaded from The Cancer Genome Atlas (TCGA) database (https://cancergenome.nih.gov/). CGGA glioma data can be downloaded from Chinese Glioma Genome Atlas (CGGA) database (http://www.cgga.org.cn/).

### Financial support and sponsorship

This work was partially supported by research grants P30CA016087, P50CA225450, P30AG066512 from the National Institute of Health (NIH). The funding body has no roles in the experiment design, collection, analysis and interpretation of data, and writing of the manuscript.

### Conflicts of interest

All authors declared that there are no conflicts of interest.

### Ethical approval and consent to participat

Not applicable.

### Consent for publication

Not applicable.

### Copyright

© The Author(s) 2022.
